# Hyperdiversity of Genes Encoding Integral Light-Harvesting Proteins in the Dinoflagellate *Symbiodinium* sp

**DOI:** 10.1371/journal.pone.0047456

**Published:** 2012-10-24

**Authors:** Lynda Boldt, David Yellowlees, William Leggat

**Affiliations:** 1 School of Pharmacy and Molecular Sciences, James Cook University, Townsville, Queensland, Australia; 2 ARC Centre of Excellence for Coral Reef Studies, James Cook University, Townsville, Queensland, Australia; 3 Comparative Genomics Centre, James Cook University, Townsville, Queensland, Australia; Arizona State University, United States of America

## Abstract

The superfamily of light-harvesting complex (LHC) proteins is comprised of proteins with diverse functions in light-harvesting and photoprotection. LHC proteins bind chlorophyll (Chl) and carotenoids and include a family of LHCs that bind Chl *a* and *c*. Dinophytes (dinoflagellates) are predominantly Chl *c* binding algal taxa, bind peridinin or fucoxanthin as the primary carotenoid, and can possess a number of LHC subfamilies. Here we report 11 LHC sequences for the chlorophyll *a*-chlorophyll *c*_2_-peridinin protein complex (acpPC) subfamily isolated from *Symbiodinium* sp. C3, an ecologically important peridinin binding dinoflagellate taxa. Phylogenetic analysis of these proteins suggests the acpPC subfamily forms at least three clades within the Chl *a/c* binding LHC family; Clade 1 clusters with rhodophyte, cryptophyte and peridinin binding dinoflagellate sequences, Clade 2 with peridinin binding dinoflagellate sequences only and Clades 3 with heterokontophytes, fucoxanthin and peridinin binding dinoflagellate sequences.

## Introduction

Light-harvesting complexes (LHC) of photosynthetic eukaryotes bind pigments essential for augmenting light capture and photoprotection. The LHC superfamily can be divided into three major groups based on the pigments bound to the protein complexes [1]. The LHCs of green plants, euglenophytes and chlorophytes bind Chl *a* and *b* (Cab; *Lhca* and *Lhcb* genes); a second group containing Chl *a* and *c* (Cac) binding LHCs is found in chromoalveolates and includes the fucoxanthin-Chl *a/c* proteins (Fcp; *Lhcf* genes); the third major group contains the Chl *a* and phycobilin binding proteins of cyanobacteria, rhodophytes (LhcaR; *Lhcr* genes) and cryptophytes (Lhc; *Lhcc* genes). Further groups within the LHC superfamily include the LI818 and LI818-like proteins [2] while more recent extensive phylogenetic analysis have identified the Lhcz proteins of cryptophytes, haptophytes and heterokonts [3] and a new family of red lineage chlorophyll *a/b*-like proteins [4].

Plants and chlorophytes have been extensively studied and contain two major Chl *a/b* binding proteins, LHCI and LHCII. These are primarily associated with photosystem I (PSI) and photosystem II (PSII) respectively. The functional unit of the green plant LHCII is a trimer, each monomer of which possesses three membrane-spanning α-helices with two short amphiphilic α-helices on the lumenal side; one at the C-terminal end [5] and the second between helices I and II [6], [7]. In the green plant LHCII model a minimum of 14 Chls (eight Chl *a* and six Chl *b*) and four carotenoids bind to the polypeptide [6], [7].

While Chl *a/b*, Chl *a/c*, and Chl *a* phycobilin binding proteins demonstrate considerable sequence divergence, the highly conserved LHCII polypeptide sequences of green plants share homology with LHCI of Chl *a* binding rhodophytes [8], [9] and the major Chl *a/c* binding proteins of dinoflagellates [10], [11]. Given this, rhodophytes and chromoalveolates might be expected to display a similar LHC structure to that of the green plant LHCII [1], [5].

To date, no rhodophyte LHCs associated with PSII have been identified, instead phycobilisomes are used as the major PSII antennae [12], [13]. Rhodophyte LHCI [8], [9] as well as chromophyte and dinoflagellate LHCs [10], [14], [15] contain the Chl-binding and stabilization residues identified in green plant LHCII and possess three membrane spanning helices [15] but do not contain the short amphiphillic α-helix on the lumenal side identified by Kühlbrandt, Wang, and Fujiyoshi [5] in green plants. The greatest variation between Chl *a/b*, Chl *a/c* and Chl *a* binding proteins is evident in helix II and the interhelical connectors; for example, in green plants seven residues separate the two Chl-binding residues (*Q* and *E*) in helix II [5], while eight amino acids separate the binding residues in the Chl *a/c* proteins and those that bind phycobilin [9].

The Chl *a/c* containing dinoflagellates are an ecologically important group of unicellular eukaryotes found in fresh and marine waters as free-living organisms, or in symbiosis with a variety of marine invertebrates and protists [16]. Studies of photoautotrophic dinoflagellates provide unique insights into plastid acquisition as they have undergone multiple chloroplastic acquisitions and subsequent gene transfer and genome reorganization, and have evolved unique protein import pathways for chloroplast-targeted proteins [17], [18]. Dinoflagellate chloroplasts are similar to those in Euglenophyta in that they are surrounded by at least three membranes. This multi-membrane arrangement has necessitated novel strategies for the importation of proteins into the chloroplast. For example, *Euglena gracilis* LHCs are initially expressed in the cytosol as polyproteins containing multiple LHC polypeptides. These are transported to the chloroplast where specific proteases cleave the polypeptide into individual mature LHC polypeptides [19], [20]. *E. gracilis* LHC polyproteins contain either nearly identical repeating polypeptides, pairs of divergent polypeptides, or complex divergent polypeptides [20]. Each LHC polypeptide is separated from the next by a decapeptide linker that contains the protease cleavage site [20]. Similarly, the dinoflagellate *Amphidinium carterae* encodes integral peridinin light-harvesting proteins as polyproteins containing up to 10 different but closely related polypeptides [11]. Circumstantial evidence also exists for other nuclear encoded chloroplast proteins being synthesized as polyproteins, for example the nuclear encoded form II Rubisco in *Symbiodinium* [21]. In addition, a subset of dinoflagellate nuclear genes can be arranged in tandem repeats and expressed on a single polycistronic mRNA, with a 5′ splice leader (SL) sequence added by *trans-*splicing [22], [23], [24]. The process of SL *trans-*splicing converts polycistronic messages into individual monocistronic mRNAs before translation [22] and in dinoflagellates polycistronic mRNA appears to contain repeated coding sequences for the same gene [24]. In euglenophytes and dinoflagellates the import of nuclear encoded proteins into the chloroplast requires mechanisms to navigate the three or more membranes surrounding the plastid [25], [26].

One group of dinoflagellates of particular ecological importance are those belonging to the genus *Symbiodinium*. These are symbiotic with reef-building corals and a variety of other marine invertebrates and encode LHCs that bind Chl *a/c* and peridinin as the primary carotenoid. Peridinin is unique to dinoflagellates [27] and is associated with two unrelated LHCs in *Symbiodinium*; the peridinin-Chl *a* protein complexes (PCP) located on the periphery of the thylakoid membranes, and the integral Chl *a*-Chl *c*_2_-peridinin protein complex (acpPC; previously ACP or *i*PCP; proposed *Lhcd* genes [28]). PCP is a soluble protein complex with no sequence similarity to other known LHCs [29]. There is a considerable body of literature on PCP [30], [31], [32], [33], [34], [35], [36], [37] and the x-ray structure has been determined [38]. In comparison, the integral protein complex acpPC does have sequence similarity with known LHCs and is part of the Chl *a/c* subfamily of LHCs [10].

Here we report a number of LHC sequences from the dinoflagellate *Symbiodinium* sp. (C3 *sensu* [39]). Phylogenetic analysis and structural comparison of a putative acpPC subfamily of genes from *Symbiodinium* demonstrates the diversity of LHC proteins in this organism. Interestingly, *Symbiodinium* sequences similar to those of the Chl *a* binding rhodophytes and Fcp binding chromophytes are evident within the one organism, although none group with the LI818 or LI818-like sequences. In addition, we demonstrate a subset of *Symbiodinium* cDNA sequences encoding polypeptides suggesting *Symbiodinium*, like *A. carterae* and *E. gracilis,* either translates LHC genes as polyproteins or alternatively, repeats of specific LHC genes are expressed on a single polycistronic mRNA transcript.

## Materials and Methods

### EST Sequencing

Partial dinoflagellate gene sequences were obtained from an Expressed Sequence Tag (EST) library of *Symbiodinium* sp. C3 [40]. Specific primers (Sigma Genosys, Australia) were designed to putative acpPC sequences and DNA amplification was performed with Platinum® Taq DNA polymerase (Invitrogen, USA) using the following conditions: an initial cycle of 2 min at 94°C, followed by 35 cycles of 20 s at 94°C, 20 s at 56°C and 2 min at 72°C, finally 10 min at 72°C and a holding temperature of 4°C. Amplified DNA was purified using QIAquick PCR Purification Kit (Qiagen, USA) prior to ligation into pGEM-T Vector System (Promega, USA) and used to transform One Shot® TOP10 Competent Cells (Invitrogen, USA). Cells were grown overnight at 37°C on LB agar treated with ampicillin. Sterile LB aliquots were inoculated with single bacterial colonies containing putative acpPC sequence inserts and the cultures grown overnight at 37°C while shaking (260 rpm). Cells were collected by centrifugation (1 min and 10,000×*g*) and plasmids purified using UltraClean™ 6 Minute Mini Plasmid Kit (Mo Bio Laboratories, Inc, USA).

Direct sequencing of EST library glycerol stocks of single bacterial colonies containing acpPC sequence inserts was also performed to confirm full insert coverage. Glycerol stocks were streaked onto LB agar with ampicillin and grown following the procedure outlined above. All clones were sequenced at the Australian Genome Research Facility (AGRF) and resulting nucleotide sequences trimmed and assembled using Lasergene® v8.0 (DNAStar Inc, Madison, USA). *Symbiodinium* acpPC sequences were identified based on transcript (blastN) and protein (blastX) similarity searches using NCBI databases and gene specific primers designed to the resulting consensus sequences using DNAStar Lasergene® v8.0 software PrimerSelect (USA).

### 5′ Sequencing

To obtain 5′ regions for the acpPC sequences amplified from the C3 EST library a 22 nucleotide 5′ *trans-*spliced leader (SL) sequence [22], [23] was used in combination with multiple gene specific primers and cDNA template transcribed from freshly extracted *Symbiodinium* sp. C3 RNA. RNA extraction and cDNA synthesis was performed on *Symbiodinium* as per Boldt, Yellowlees, and Leggat [41]. *Symbiodinium* cells were harvested from *Acropora aspera* branches collected from the reef flat at Heron Island (Great Barrier Reef (23°33′S, 151°54′E) in April 2009 and frozen in liquid nitrogen.

Sequences were confirmed by amplification using high fidelity AccuPrime™ *Pfx* SuperMix (Invitrogen, USA) and 200 nM final concentration of each primer. The final 25 µL reaction mixture was amplified on a MultiGene Thermal Cycler (Labnet International Inc, NJ, USA) using the following conditions: an initial cycle of 5 min at 95°C, followed by 35 cycles of 15 s at 95°C, 30 s at 60°C and 90 s at 68°C, finally 10 min at 72°C and a holding temperature of 4°C. Zero Blunt^®^ TOPO^®^ PCR Cloning Kit for sequencing was used to insert amplified blunt-end products into the plasmid vector pCR^®^4Blunt-TOPO^®^ and transformed into One Shot® TOP10 Cells (Invitrogen, USA) according to manufacturer’s protocol. A second set of sequences were obtained following template purification using QIAquick PCR Purification Kit (Qiagen, USA), addition of dATP overhangs, ligation into pGEM-T Vector and transformation into NM522 cells. A third and final set of sequences obtained following template amplification with Promega GoTaq® Flexi DNA polymerase (Promega Corporation, Madison, WI, USA) using the following conditions: an initial cycle of 2 min at 95°C, followed by 35 cycles of 30 s at 95°C, 30 s at 60°C and 2 min at 72°C, finally 10 min at 72°C and a holding temperature of 4°C. Purified PCR product (QIAquick PCR Purification Kit, Qiagen, USA) was directly ligated into pGEM-T Vector and transformed into NM522 cells. Each sequencing method either provided new sequence data or confirmed data already obtained. Following plasmid purification (PureLink™ HQ Mini Plasmid Purification Kit, Invitrogen, USA) multiple clones for each gene were sequenced at the AGRF to confirm sequencing results.

### Northern Blot Analysis for *Symbiodinium* acpPC

Transcript size of five different acpPC genes (acpPCSym_1, _4, _8, _13 and _15) was determined using northern blot analysis. Total RNA from *Symbiodinium* C3 and C1 were extracted from 100 mg of crushed *A. aspera* skeleton (C3 cells) or 6–10×10^6^ cultured C1 cells using an RNeasy Plant Mini Kit (Qiagen, USA). Cultured C1 *Symbiodinium* (CCMP 2466, USA) were lysed twice for 20 s at 4.0 ms^−1^ in Lysing Matrix D tubes (MP Biomedicals, Australia) containing 450 µL of RLT Buffer (Qiagen, USA) on a FastPrep®-24 Instrument (MP Biomedicals, Australia). RNA precipitations were performed using 100% ethanol and RNAse free sodium acetate and water. Two and one half µg of total RNA was separated on a 1.2% formaldehyde agarose gel at 50 volts for 4 h and subsequently transferred to Amersham Hybond™-N+ (GE Healthcare Life Science, UK) membranes according to Sambrook and Russell [42]. Membranes were crossed linked for 90 s in a microwave (1100 watts) and prehybridized for 2–6 h at 58–60°C in Denhardts buffer (5× SSC, 5× Denhardt’s solution, 0.5% w/v SDS). Probes designed to five *Symbiodinium* acpPC genes (acpPCSym_1, _4, _8, _13 and _15) using DNASTAR Lasergene® v8.0 PrimerSelect (USA) were labeled using dATP 5′ – [α-^32^P] (PerkenElmer, USA) and Prime-A-Gene® Labelling System (Promega Corporation, Madison WI, USA). After hybridization for 20 h at 58–60°C membranes were washed for 5 min in 2× SSC/0.1% SDS at room temperature, 15 min in 1× SSC/0.1% SDS at room temperature then 10 min in 1× SSC/0.1% SDS at 55°C and air-dried on blotting paper. Saran wrapped membranes were exposed to a storage phosphor screen (Molecular Dynamics, GE Healthcare Life Sciences, UK) for 48 - 96 h and visualized using a PhosphorImager Storm 860 by Molecular Dynamics (GE Healthcare Life Science, UK).

### Phylogenetic Analysis

LHC sequences for 35 species belonging to 33 genera of green plants, chlorophytes, chlorarachniophytes, prasinophytes, heterokontophytes, a rhodophyte, euglenophyte, cryptophyte, haptophyte and dinophytes (see Table S1 in the Supplementary Material) were aligned with acpPC sequences from *Symbiodinium* sp. C3. *Symbiodinium* sequences encoding multiple copies of the membrane spanning helices (acpPCSym_1, _5, _8, _10, _12, _13 and _17) were divided into individual units comprised of helices I and II, and helices III and IV when present. Alignments of protein sequences were constructed in BioEdit Sequence Alignment Editor [43] using ClustalW Multiple Alignment [44] using default settings. Alignment refinement was manually performed using Jalview v2.4 [45] based on the highly conserved Chl-binding residues and stroma localized motifs proposed by Kühlbrandt, Wang, and Fujiyoshi [5] and Tan et al. [9] as guides to helix location. Regions of high divergence were excluded from the analysis because of the ambiguity required to align these regions with only the first three helices, and amphiphilic α-helix when present, used in analysis to maximise position homology. Phylogenetic analysis was performed using PhyML for maximum likelihood [46] and MrBayes for Bayesian inference [47]. Using ProtTest v2.1 [48], the LG + Alpha + Proportion Invariant + Frequencies (LG+I+G+F) model of protein evolution was determined as the best fit model for the final alignment data. LG [49], a relatively new amino acid replacement matrix, is not a candidate model to determine Bayesian inference thus the WAG matrix [50] was selected for MrBayes analysis. The most likely topology was calculated based on Sh-like branch support. For the Bayesian inference, two runs with 10 million generations each were calculated with topologies saved at each 1,000 generations. One fourth of the 10,000 topologies were discarded as burn in, and the remaining used to calculate the posterior probability.

Hydrophobicity plots generated with DNASTAR Lasergene® v8.0 Protean program (USA), using a Kyte-Doolittle scale of 20 residues, in conjunction with the hidden Markov model-based program HMM [51], PolyPhobius [52] and ChloroP [53] were used to identify potential membrane-spanning regions, signal peptides and chloroplast transit peptides in acpPC sequences. Sequence logos of helices I - IV were generated using WebLogo [54] from the sequence data used to generate phylogenetic analysis.

### Accession Numbers

Sequence data from this article can be found in the EMBL/GenBank data libraries under the accession numbers FN646412-FN646425.

## Results

### Phylogenetic Analysis

**Figure 1:**
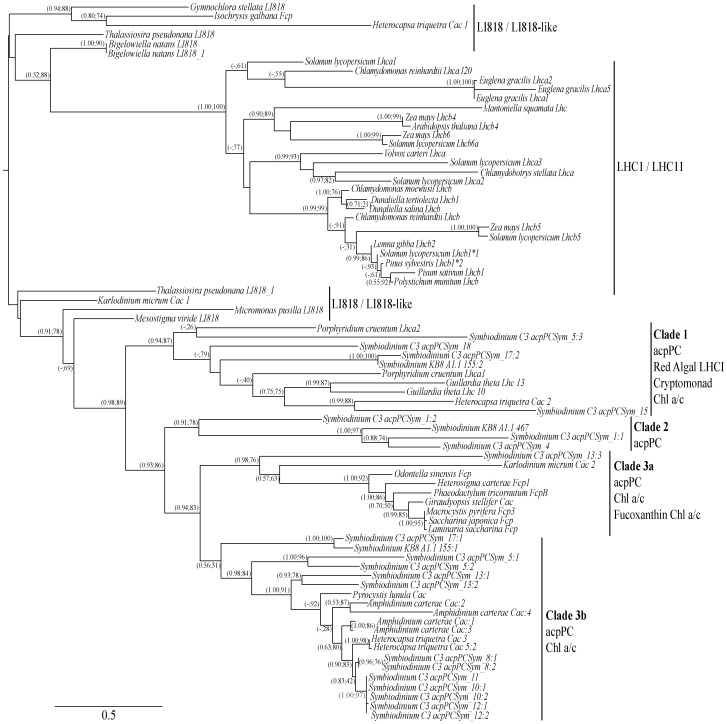
Phylogenetic analysis of *Symbiodinium* sp. C3 acpPC sequences with LHCs from green plants, chlorophytes, chlorarachniophytes, prasinophytes, heterokontophytes, a rhodophyte, euglenophyte, cryptophyte, haptophyte and dinophytes. A maximum likelihood tree rooted at the mid-point is shown with the support values for MrBayes (posterior probability) followed by PHYML included at the nodes. A total of 78 sequences with 97 amino acid sites (including gaps) were analyzed. The Chl *a/b* binding protein complexes associated with PSI (*Lhca*) and PSII (*Lhcb*) in green plants, chlorophytes and euglenophytes cluster together while the Chl *a/c* binding protein complexes form a second cluster which includes the Fcp sequences of heterokontophytes, *Lhca*s of a rhodophyte and LHCs of a cryptophyte. The LI818 and LI818-like sequences group separately but include a haptophyte Fcp sequence and two dinophyte Chl *a/c* sequences. Within the Chl a/c cluster are three distinct clades: Clade 1 containing four *Symbiodinium* sp. C3 acpPC sequences and LHCs from a rhodophyte, cryptophyte, and two peridinin-binding dinophytes, *Heterocapsa triquetra* and *Symbiodinium* type A1.1; Clade 2 contains *Symbiodinium* sequences; Clade 3 is divided into two clusters, a Chl *a/c* and Fcp cluster (3a), and a *Symbiodinium* sp. C3 acpPC and Chl *a/c* cluster (3b). Sequence data obtained from multiple databases is available in the online Supplementary Material (Table S1).

**Figure 2:**
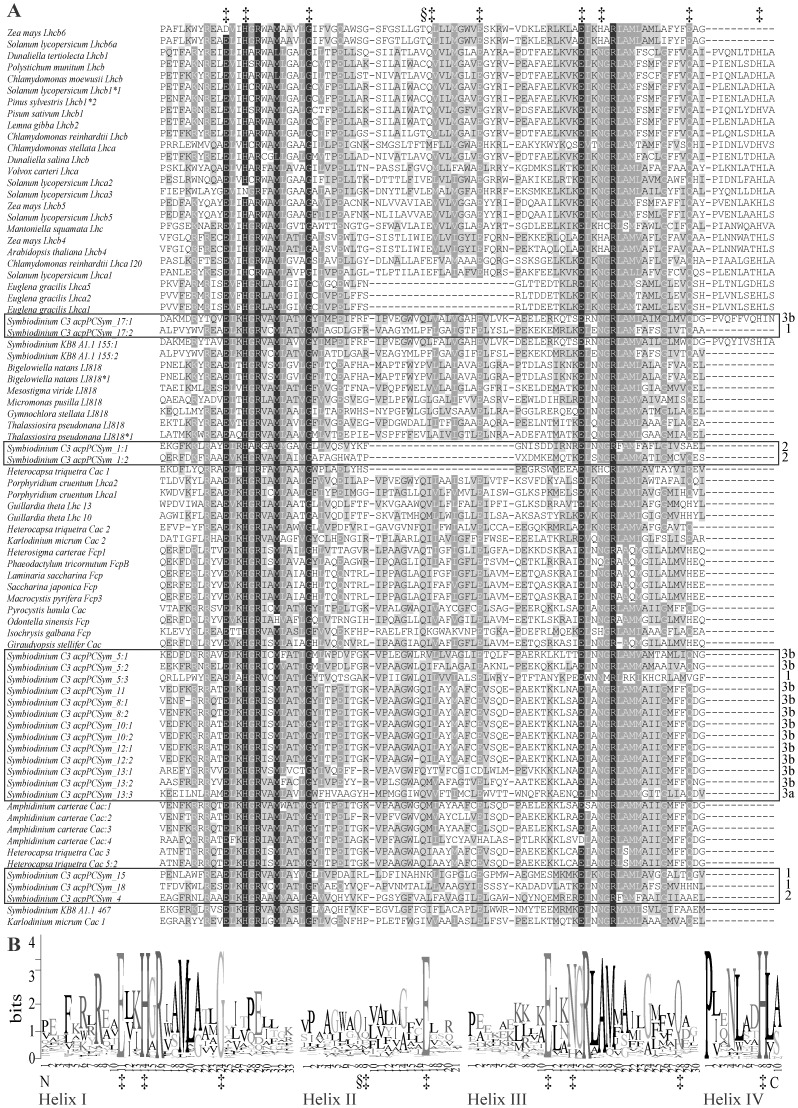
Alignment of *Symbiodinium* LHC with other LHC representatives. (A) Amino acid alignment of membrane-spanning helices for 78 sequences used to determine *Symbiodinium* sp. C3 integral LHC phylogeny. Black shading represents 99% conservation, dark grey 70% and light grey 35%. *Symbiodinium* sp. C3 acpPC sequences are boxed and the side numbering (1, 2, 3a and 3b) refers to the three distinct clades within the Chl *a/c* binding cluster. Clade 1 acpPC sequences cluster with a rhodophyte, cryptophyte, and dinophytes possessing three transmembrane helices. Clade 2 is a dinophyte cluster and includes acpPCSym_1 which lacks helix II. Clade 3a is a Chl *a/c* and Fcp cluster while Clade 3b is a second cluster of dinophytes possessing three transmembrane helices with the exception of acpPCSym_17, which has a fourth amphiphilic α-helix. (B) Amino acid sequence logo plot of LHC membrane-spanning helices I – IV. Position 1 denotes the start of a helix and the higher the bits score the greater the consensus across the data. In both the alignment and logo plot a manually inserted gap separates each helix and the nine proposed Chl-binding residues, identified in the green plant LHCII model [5], are listed and marked (‡): Helix I: E-11, H-14, G-24; Helix II: Q-8, Q-9 E-17; Helix III: E-11, N-14, Q-28; Helix IV: H-8. In helix II, *Q* at position 8 (§) and 9 (‡) demonstrates the varied spacing between the two Chl-binding residues, with Chl *a/b* binding proteins separated by seven residues and Chl *a/c* binding proteins separated by eight. Two arginine residues (Helix I: R-16; Helix III: R-16) are proposed to play a stabilization role in helices I and III.

Expressed sequence tags (ESTs) for *Symbiodinium* sp. C3 isolated from *Acropora aspera* [40] were used to obtain partial sequences for putative acpPC genes. ESTs for 33 clones were sequenced and assembled based on a minimum match percentage of 98%. High fidelity amplification of the resulting 14 acpPC sequences provided 5′ regions for nine, with the remaining five sequences extended but lacking full sequence coverage (table 1). These sequences were named acpPCSym_1, 3–5, 8–15, 17 and 18. *Symbiodinium* acpPC sequences, 54 LHC protein sequences for green plants, chlorophytes, chlorarachniophytes, prasinophytes, heterokontophytes, a euglenophyte, rhodophyte, cryptophyte, haptophyte and dinophytes including EST sequence data for *Symbiodinium* clade A1.1 [55] were aligned and the membrane-spanning sequences used for phylogenetic analysis (fig. 1). *Symbiodinium* acpPC sequences encoding repeats of the three transmembrane helices in single LHC protein were treated as multiple sequences and numbered individually using a single colon to separate the sequence name from the transmembrane helices group in order from the 5′ end (for example, acpPCSym_5∶1, _5∶2 and _5∶3, each contain helices I, II and III) (figs. 1 and 2a). These results demonstrate *Symbiodinium* sp. C3 encode a diversity of integral LHC proteins ranging in size from 18 - 65 kDa and importantly, LHC sequences similar to those of the Chl *a* binding rhodophytes and Fcp binding heterokontophytes are evident within this one species of dinoflagellate (fig. 1).

**Table 1:** *Symbiodinium* sp. C3 chlorophyll *a*-chlorophyll *c*_2_-peridinin characteristics.

LHC name	Complete sequence coverage	cDNA size (bp)	Protein size (aa) andmass (kDa)	Polypeptide number-size(s) (aa)/mass(es) (kDa)	SPLR motif	Predicted chloroplast transit peptide/signal peptide/transmembrane helices	Membrane spanning helix organisation
acpPCSym_15	yes	932	276 aa/28842 kDa	1	no	no/no/yes	H1-H2^b^-H3
acpPCSym_18	no	785	231 aa/24007 kDa	1	no	yes/no/yes	H1-H2^b^-H3
acpPCSym_1	yes	1376	440 aa/45941 kDa	1	no	yes/yes/yes	H1-H3-H1-H3
acpPCSym_4	yes	1131	292 aa/28979 kDa	1	no	yes/yes/yes	H1-H2^b^-H3
acpPCSym_5	no	1836	592 aa/65482 kDa	1	no	no/no/yes	H1-H2^b^-H3-H1-H2-H3-H1-H2-H3
acpPCSym_8	yes	1403	433 aa/44849 kDa	2–173 aa/18357 kDa	yes x2	yes/yes/yes	H1-H2-H3-H1-H2-H3
acpPCSym_10	yes	1520	468 aa/49364 kDa	2–173 aa/18451 kDa	yes x2	yes/no/yes	H1-H2-H3-H1-H2-H3
acpPCSym_11	yes	888	259 aa/26742 kDa	1–173 aa/18424 kDa	yes x1	yes/yes/no	H1-H2-H3
acpPCSym_12	yes	1371	441 aa/45922 kDa	2–173 aa/18439 kDa, 173 aa/18496 kDa	yes x2	yes/yes/yes	H1-H2-H3-H1-H2-H3
acpPCSym_13	yes	2097	681 aa/73911 kDa	3–192 aa/21273 kDa, 178 aa/19587 kDa, 210 aa/22949 kDa	yes x2	yes/yes/yes	H1-H2^b^-H3-H1-H2-H3-H1-H2-H3
acpPCSym_17	yes	1545	494 aa/53819 kDa	1	no	yes/yes/yes	H1-H2-H3-H4-H1-H2^b^-H3
acpPCSym_3^c^	no	844	273 aa/29889 kDa	2- incomplete	yes x2	no/no/yes	H3-H1-H2-H3
acpPCSym_9^c^	no	1039	319 aa/35093 kDa	1	no	no/no/yes	H3-H2-H3-H1
acpPCSym_14^c^	no	1570	523 aa/56719 kDa	2 - incomplete	no	no/no/yes	H2-H3-H1-H2-H3-H1-H3

a Sequences marked as containing one polypeptide are monomeric proteins while acpPCSym_8, _10, and _12 are bipartite polypeptides. The sequence for acpPCSym_13 encodes a tripartite polypeptide and the polypeptide at the 3′ end lacks the C-terminal SPLR motif present in the first two polypeptides.

b Variation to Chl-binding residues in helix II from residues (*Q* and *E*) identified in the green plant LHCII three transmembrane and amphiphilic α-helix protein present.

c Sequences were not included in phylogenetic analysis.

Phylogenetic analysis of the sequences for the LHC membrane-spanning helices demonstrates the distinct separation of Chl *a/b* and Chl *a/c* binding protein lineages (fig. 1). The Chl *a/b* lineage comprises LHCI and LHCII sequences from green plants, chlorophytes, a prasinophyte and euglenophyte. The Chl *a/c* lineage includes Chl *a/c* and Fcp binding proteins from heterokontophytes and dinophytes, a rhodophyte and cryptophyte and the putative dinoflagellate acpPC family of sequences. This separation of LHC proteins into two distinct lineages corresponds with the presence or absence of chlorophyll *b* in light-harvesting and has previously been well documented [15], [56].

Within the lineage of Chl *a/c* binding proteins, three distinct clades can be found (fig. 1). Clade 1 includes the rhodophyte and cryptophyte sequences, two dinoflagellate Chl *a/c* sequences and four acpPC sequences (acpPCSym_5∶3, _17∶2, _15 and _18). Clade 2, contains three *Symbiodinium* sequences. acpPCSym_1 and _4 sequences encode monomers of 46 kDa and 29 kDa respectively with chloroplastic leader sequences and the N-terminal four-residue phenylalanine based motif features in the transit peptide.

**Figure 3:**
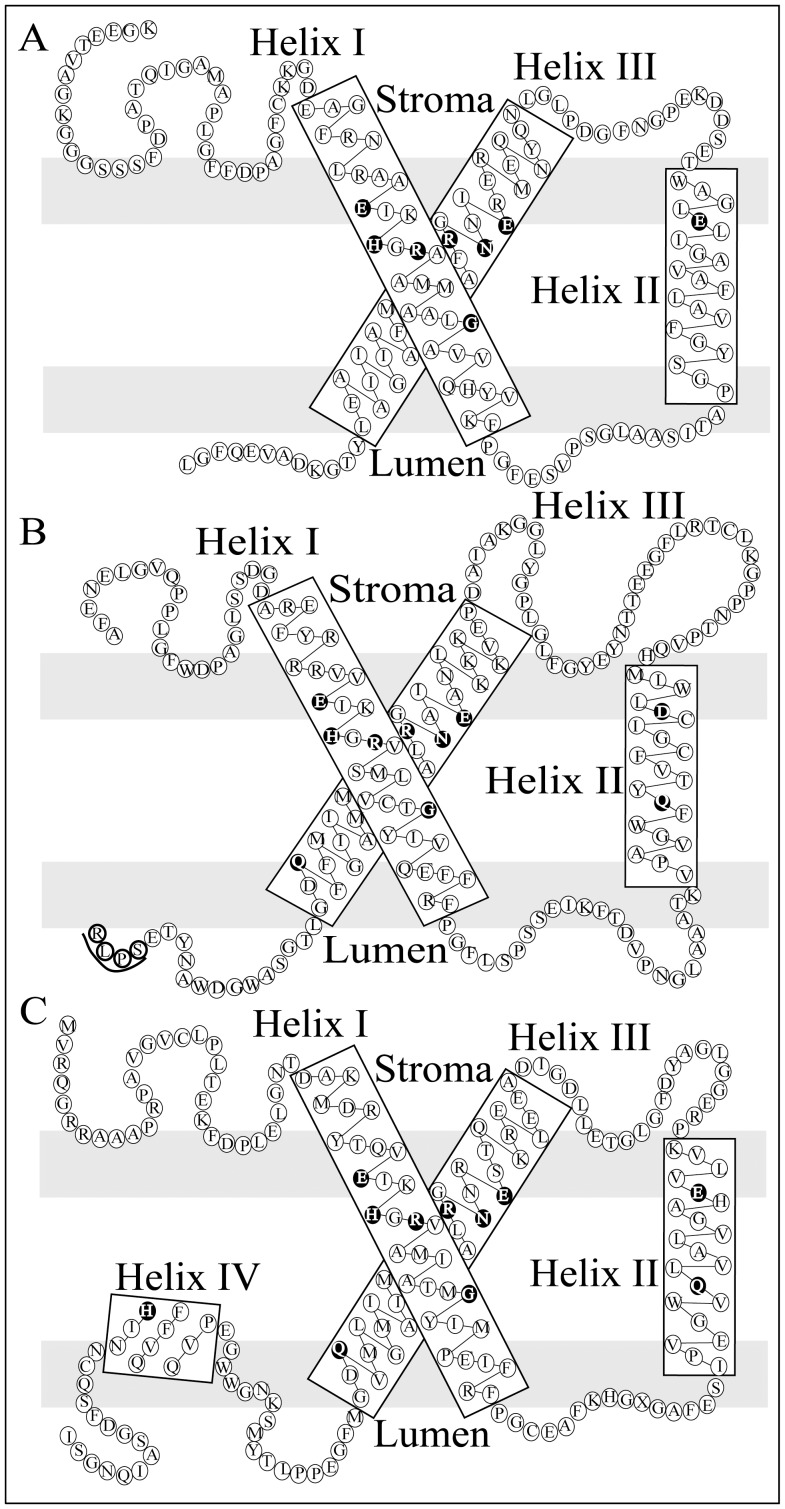
Schematic representation of the different *Symbiodinium* transmembrane domains. (A) Schematic of the three transmembrane polypeptide encoded by acpPCSym_4 in *Symbiodinium* sp. C3. acpPCSym_4 clusters with dinophyte LHCs and helix II and helix III contain a variation to one Chl-binding residue proposed in the Kühlbrandt, Wang, and Fujiyoshi [5] model for green plants. (B) Schematic of the three transmembrane polypeptide acpPCSym_13∶1 in *Symbiodinium* C3. acpPCSym_13∶1 clusters with Chl *a/c* LHCs and encodes a proposed proteolytic cleavage site for mature polypeptides (SPLR) on the lumenal side and C-terminal to helix III. (C) Schematic of the three transmembrane and fourth amphiphilic α-helix polypeptide encoded by acpPCSym_17∶1 in *Symbiodinium* C3. acpPCSym_17∶1 clusters with Chl *a/c* LHCs, includes helix IV, however helix II has eight amino acid residues between the two Chl-binding residues rather than the seven evident in helix II of green plants. acpPCSym_17∶2 encodes a second set of helices I, II and III but not helix IV. Schematics are based on the LHCII model of Kühlbrandt, Wang, and Fujiyoshi [5] and residues identified as Chl-binding or involved in helix stabilization are shown with a black background.

The final cluster within the Chl *a/c* lineage, Clade 3, comprises two clusters; Clade 3a includes heterokontophyte Fcp sequences, Chl *a/c* sequences and one acpPC sequence, while Clade 3b is a dinoflagellate specific clade and contains sequences from four genera of dinoflagellate, including twelve acpPCSym sequences. Each dinoflagellate genera in Clade 3b encode LHCs with a C-terminal SPLR motif after helix III (fig. 3b). In *A. carterae* the Chl *a/c* polyprotein contains a protease cleavage site at the C-terminal arginine residue of the SPLR motif which generates at least five separate mature LHC polypeptides [11]. Four of the *Symbiodinium* acpPC sequences are potentially polyproteins containing multiple LHC polypeptides and C-terminal SPLR motifs (acpPCSym_8, _10, _12, _13). Three acpPC sequences acpPCSym_8, _10, and _12 encode bipartite polypeptides each of 18.4 kDa while acpPCSym_13 encodes tripartite polypeptides of 21 kDa, 19.5 kDa and 23 kDa (table 1). The two 5′ end polypeptides in acpPCSym_13 (_13∶1 and _13∶2) contain the C-terminal SPLR motif while the third (_13∶3) does not. A fifth sequence (acpPCSym_11) contains the C-terminal SPLR motif, but encodes only one LHC polypeptide of 18.4 kDa. This monomer has high identity at the protein level (93–95%) and cDNA level (79–98%) with polypeptides from acpPCSym_8, _10 and _12 (table 2). In comparison, the two polypeptides containing SPLR within acpPCSym_13 have only 57% identity at the protein level with acpPCSym_11, and polypeptide three is only 23% (table 2).

**Table 2:** Comparison of the cDNA and amino acid sequences in the *Symbiodinium* sp. C3 chlorophyll *a*-chlorophyll *c_2_*-peridinin light-harvesting complex.^a^.

LHC	acpPCSym	acpPCSym	acpPCSym	acpPCSym	acpPCSym	acpPCSym	acpPCSym	acpPCSym	acpPCSym	acpPCSym
Name	_11	_8∶1	_8∶2	_10∶1	_10∶2	_12∶1	_12∶2	_13∶1	_13∶2	_13∶3
acpPCSym	23	24	24	23	23	23	23	21	23	100
_13∶3	23	36	36	37	37	38	38	36	35	100
acpPCSym	56	57	57	57	57	57	55	54	100	
_13∶2	37	58	57	59	58	58	57	57	100	
acpPCSym	57	55	55	57	57	57	56	100		
_13∶1	36	58	57	58	58	58	58	100		
acpPCSym	97	90	91	95	95	97	100			
_12∶2	96	79	80	94	92	96	100			
acpPCSym	99	92	93	97	97	100				
_12∶1	98	79	80	93	92	100				
acpPCSym	97	93	94	100	100					
_10∶2	93	79	79	97	100					
acpPCSym	97	93	94	100						
_10∶1	95	80	80	100						
acpPCSym	93	99	100							
_8∶2	80	97	100							
acpPCSym	93	100								
_8∶1	79	100								
acpPCSym	100									
_11	100									

a Comparisons are made at the percent identity level. The upper number refers to protein identity while the percent identity in the cDNA is beneath.

acpPCSym_11 encodes a monomeric protein, acpPCSym_8, _10 and _12 encode bipartite polypeptides. Individual polypeptides in the polyproteins are numerically identified for example, acpPCSym_13∶1 is the polypeptide at the N-terminal end of the tripartite polyprotein. Each polypeptide has the C-terminus motif SPLR with the exception of acpPCSym_13∶3.

Two transcripts (acpPCSym_5 and _17) do not contain the SPLR motif but encode multiple copies of the three transmembrane helices found in single LHC proteins. The transcript for acpPCSym_5 encodes a 65.5 kDa protein with nine membrane-spanning regions, but lacks full coverage in the 5′ region. The complete sequence for acpPCSym_17 encodes a 54 kDa protein with seven membrane-spanning regions and includes a chloroplastic leader sequence (table 1) containing a transit peptide with an N-terminal four-residue phenylalanine based motif [18]. The lack of a canonical C-terminal SPLR like motif in acpPCSym_5 and _17 suggests that the mature protein is either a monomer or has a proteolytic cleavage site different from the *A. cartera*e, *Pyrocystis lunula, Heterocapsa triquetra* and acpPC sequences containing SPLR.

### Transmembrane Topology

Similar to rhodophyte, cryptophyte and heterokontophyte LHCs, acpPC sequences generally contain three transmembrane helices (Helix I, Helix II and Helix III; fig. 2a). In comparison, green plant and chlorophyte LHCs generally contain three transmembrane helices and a fourth amphiphilic α-helix. In *Symbiodinium* sp. C3 acpPC sequences the structural features and Chl-binding residues identified in the green plant LHCII model are generally present (figs. 2a and 2b). Twelve of the acpPC sequences code for eight amino acid residues between the *Q* and *E* Chl-binding residues in helix II while six lack one of these residues (table 1 and fig. 2a). Five proteins have a non-conservative substitution of the *Q* residue (fig. 3a), similar non-conservative substitutions can be found in the dinoflagellates *Symbiodinium* KB8 A1.1 and *Karlodinium micrum* and the green alga *Chlamydobotrys stellata,* while acpPCSym_13 has a conservative substitution in the second Chl-binding residue with *D* replacing *E* (fig. 3b). The conserved residue substitution in acpPCSym_13 is only evident in the first group of membrane-spanning helices (acpPCSym_13∶1), the second (_13∶2) and third (_13∶3) group contain *Q* and *E* separated by eight residues. A similar substitution pattern is evident in the acpPCSym_5 monomer (table 1).

In addition to variations of specific Chl-binding residues there are examples of significant divergence in the secondary structure of *Symbiodinium* LHCs. acpPCSym_1 is noticeably different from the other acpPC sequences in that it lacks helix II (table 1 and fig. 2a) and encodes membrane-spanning helices (acpPCSym_1∶1, _1∶2) with the highly conserved helices I and III repeated. The absence of helix II sequence also occurs in *H. triquetra* Cac_1 that clusters with LI818 and LI818-like genes (figs. 1 and 2a), and in some *E. gracilis* membrane-spanning helices (fig. 2a). Within Clade 3b (fig. 1), acpPCSym_17∶1 exhibits the canonical structure of a three transmembrane helix protein with a amphiphilic α-helix (figs. 2a and 3c) common amongst Chl *a/b* lineage LHCs rather than only three transmembrane helix protein common within the Chl *a/c* binding lineage. The presence of helix IV is not evident in proteins within the Chl *a/c* lineage with the exception of *Symbiodinium* KB8 A1.1_155∶1.

### Polycistronic Transcript Variation

**Figure 4:**
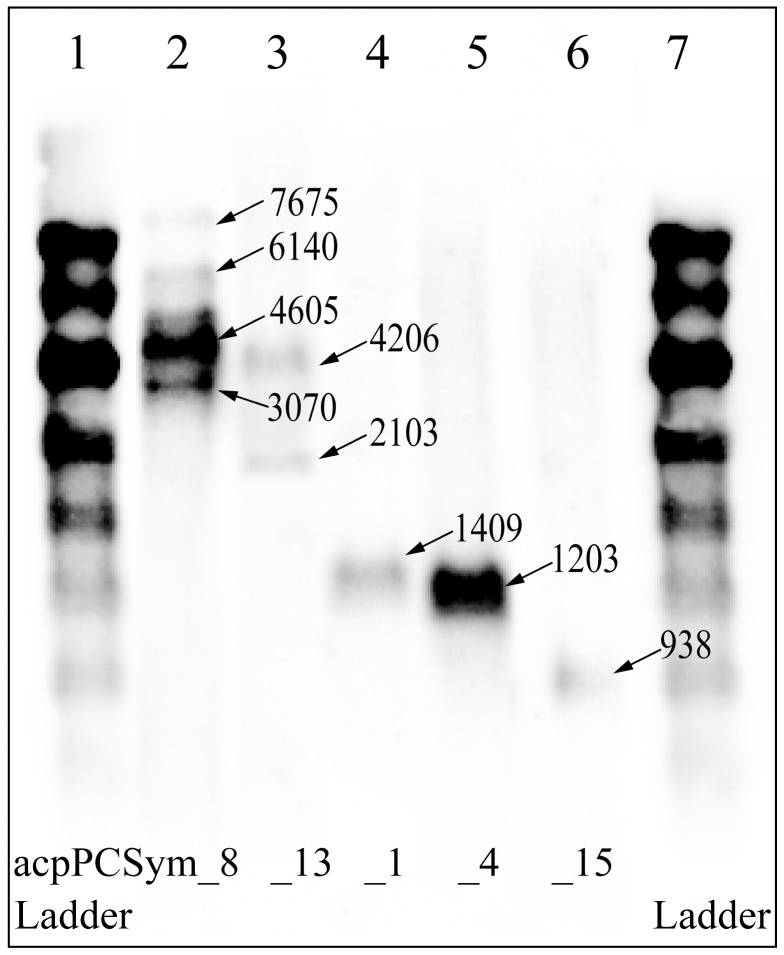
Northern blot analysis of cultured *Symbiodinium* sp. C1 RNA. The blot was probed with sequence specific PCR product for *Symbiodinium* acpPCSym_8 (lane 2), acpPCSym_13 (lane 3), acpPCSym_1 (lane 4), acpPCSym_4 (lane 5) and acpPCSym_15 (lane 6) labeled using dATP 5′ – [α-^32^P]. Equal quantities (2.5 µg) of RNA from *Symbiodinium* sp. C1 were applied. Lanes 1 and 7 contain RNA standard probed with lambda DNA.

*Symbiodinium* sp. C3 encodes transcripts containing one, two or three LHC polypeptides. To determine if larger transcripts are present in *Symbiodinium*, LHC transcript size was examined by northern blot analysis on RNA from freshly isolated *Symbiodinium* sp. C3 (see fig. S1 in the Supplementary Material) and the closely related cultured *Symbiodinium* sp. C1 (fig. 4). Probes used represented the diversity of LHC transcripts including the Chl *a/c* and Fcp-like acpPCSym_8 and _13 (Clade 3b), which contain multiple polypeptides, and acpPCSym_1, _4 (Clade 2) and _15 (Clade 1), which represent the LHC group with transcripts containing monomers. The probes hybridized strongly with cultured C1 RNA but there was weak hybridization of the same probes to C3, possibly due to coral RNA contribution within the sample. Probes for acpPCSym_1, acpPCSym_4 and acpPCSym_15 hybridized to RNA of 1.4 kb, 1.2 kb and 938 bp corresponding to the size expected for a monocistronic polypeptide transcript (table 1). In contrast, probes for acpPCSym_8 hybridized to RNA of 3.1 kb, 4.6 kb, 6.1 kb and 7.6 kb while the acpPCSym_13 probe hybridized to RNA of 2.1 kb and 4.2 kb. Based on acpPC polypeptide size (table 1), acpPCSym_8 mRNA comprises between four and ten 519 bp repeats (173 amino acid polypeptides) and acpPSym_13 comprises three to six 576/534/630 bp repeats (192/178/210 amino acid polypeptides).

## Discussion

The *Symbiodinium* sp. C3 acpPC sequences reported here encode a diversity of light-harvesting proteins within the Chl *a/c* binding family of LHCs (fig. 1) that exhibit similarities with both the rhodophyte Chl *a* binding proteins and the fucoxanthin Chl *a/c* binding proteins, but not the LI818 or LI818-like proteins. *Symbiodinium* sp. C3 acpPC sequences may share similar structural features and Chl-binding residues as those demonstrated in the LHCII sequences of Chl *a/b* binding proteins, but the position of the sequences within the Chl *a/c*, Fcp and Chl *a* grouping is strongly supported. The diversity evident in *Symbiodinium* LHCs suggests the acpPC subfamily may contain functionally distinct LHCs that enable these ecologically important dinoflagellates to efficiently utilize the varied light conditions experienced in the marine environment.

The major divisions within the phylogenetic reconstruction of LHCs in this study were similar to previously published results focusing on chlorophyte [3] and stress-induced [57] LHCs. The division of sequences into cryptomonad/red algal, Fcp/Chl *a/c*, LI818 family, and LHCI/LHCII subgroups remained clear (fig. 1), and the diversity of LHCs demonstrated in the one species of *Symbiodinium,* presented in this study, reflects findings that Chl *a/c* containing algae possess LHCs from different subfamilies and lineages. Plastid genome sequencing of *Emiliania huxleyi* indicates organelles of haptophytes, heterokontophytes and cryptophytes are closely related [58], and genes from both red and green algal sources are evident in the heterokontophytes *Thalassiosira* and *Phaeodactylum* [59]. Fcp sequences from *Cyclotella cryptic* share similarity with LI818 proteins of two *Chlamydomonas* spp. and the haptophyte *Isochrysis galbana*, and one *C. cryptic* Fcp sequence shows similarity to the red algal *Porphyridium cruentum* LHCI sequences *Lhca1* and *Lhca2* [60].

The fucoxanthin-binding dinoflagellate *K. micrum* also exhibits diversity in LHCs (fig. 1, LI818/LI818-like and Clade 3a) and this may relate to the origin of plastids derived from haptophytes [61]. The tertiary derived plastids contain fucoxanthin-binding proteins in addition to those proteins remaining from the secondary dinoflagellate plastid that possess peridinin-binding proteins [62] and may be the case with acpPCSym_13∶3 which groups with *K. micrum* and other fucoxanthin – binding algae (Clade 3a) rather than acpPCSym_13∶1 and _13∶2 (Clade 3b). The presence of LHC sequences in Clades 1, 2 and 3 of other dinoflagellate ESTs supports the hypothesis that this characteristic may be widespread in these algae. Whether the degree of expression diversity seen here in *Symbiodinium* sp. C3 is partially a factor of the diverse array of experimental conditions to which the algae were exposed [40] is unknown.

Given the series of plastid acquisition and genome rearrangements in dinoflagellates [22], [23], and the evidence for lateral gene transfer [63], it is not surprising dinoflagellates encode multiple LHC genes. Many dinoflagellate genes have very high copy numbers encoding similar proteins; for example the *A. carterae* actin copy number is at least 113 [24] and the *Lingulodinium polyedrum* luciferin-binding protein gene has approximately 1,000 copies [64]. While those *Symbiodinium* LHCs that are closely related may be functionally equivalent, the diversity in sequences raises the possibility that some may be functionally different and involved in distinct light harvesting and photoprotection processes. The photoprotective strategy utilized by *Symbiodinium* remains unresolved, however it has recently been found that LHC can disassociate from PSII under high light conditions [65] with redistribution to PSI to minimise PSII overexcitation [66] in *Symbiodinium*, whether this occurs for all acpPC isoforms are involved in this are unknown. In addition to state transitions *Symbiodinium* acpPC bind the carotenoid pigments diadinoxanthin (Dn) and diatoxanthin (Dt) [67], which are functionally equivalent to the xanthophyll pigments central to terrestrial plants photoprotective mechanisms [68]. With respect to this, the lack of similarity of *Symbiodinium* LHC sequences presented here with LI818 or LI818-like proteins is interesting. The two subgroups of LI818 and LI818-like sequences in this study each contain one dinoflagellate representative: *H. triquetra* and *K. micrum* (fig. 1). Many proteins from the LI818 subfamily have a role in stress response and recent phylogenetic analysis suggests LI818 proteins originated in an ancestral Chl *a/c* containing organism [57]. In addition, the number of proteins from the LI818 family present in haptophytes and heterokontophytes suggests this family of proteins is important in the marine environment [57].

### LHCs Synthesized as Polyproteins

The use of polyproteins as a method for the importation of nuclear encoded Chl-binding proteins into algal chloroplasts has been reported previously for *Euglena* (Chl *a/b* binding protein) and the dinoflagellate *A. carterae* (Chl *a/c* binding protein): these have three or four and three chloroplast membranes respectively. Four *Symbiodinium* LHC proteins (acpPCSym_8, _10, _12 and _13) share identity with the Clade 3 LHCs and encode polypeptides within a single transcript. The SPLR motif was present in all *Symbiodinium* transcripts encoding polypeptides (acpPCSym_8, _10, _12 and _13). This is consistent with the mature LHCs being generated by cleavage at the arginine in this C-terminal motif [11]. The SPLR motif was also present at the C-terminal end of acpPCSym_11, which encodes a monomer. Its presence in this transcript and identity at the cDNA level of acpPCSym_11 with the polypeptides of acpPCP_8 (79% and 80%), _10 (95% and 93%) and _12 (98% and 96%) (table 2) raises the possibility that acpPCSym_11 represents a component of the original gene, which following gene duplication and fusion, has given rise to polyprotein transcripts. This contrasts with the tripartite polypeptides of acpPCSym_13 that have significantly less identity and may represent an older series of gene duplication/fusion events (table 2). Four other dinoflagellates, *A. carterae* [11], *P. lunula* [69], *H. triquetra* [18] and *Amphidinium tamarense* [70], also encode LHCs containing the motif SPLR.

Gene duplication is a well documented feature in dinoflagellates [71], [72], [73] which presumably has given rise to polyproteins in addition to increasing the copy number of highly expressed genes [24]. Information on the organisation of dinoflagellate genomes is limited, but there is evidence that subsets of genes are arranged in repeated tandem arrays and result in polycistronic messages carrying multiple copies of a single gene [24]. Analysis of 15 highly expressed *A. carterae* genes showed that 14 were arranged in repeated tandem arrays while only two moderately expressed genes had a similar arrangement [24]. In addition *A. carterae* has been shown to encode up to ten LHC polypeptides within the one transcript [11]. While transcripts encoding up to three LHC polypeptides were found in the screen of the *Symbiodinium* cDNA library, northern blot analysis indicates that *Symbiodinium* expresses larger transcripts. For example a probe using a portion of acpPCSym_8 that encodes two LHC polypeptides with a size of 1.54 kb hybridizes to transcripts of 3.1 kb, 4.6 kb, 6.1kb and 7.6 kb (fig. 4), corresponding to four, six, eight and ten LHC polypeptides.

The presence of LHC polyproteins in *E. gracilis* and dinoflagellates, which have acquired plastids from different algal lineages, probably represents the convergent evolution of an efficient strategy to import proteins through multiple chloroplastic membranes into secondary chloroplasts. The presence of multiple membranes around the plastid necessitates the presence of an N-terminal transit peptide for nuclear encoded chloroplast targeted proteins. In dinoflagellates these proteins possess presequences containing a signal peptide and a transit peptide of which there are two major classes, both possessing a four-residue phenylalanine based motif [18]. The 5′ regions of *Symbiodinium* sp. C3 acpPC sequences, for which we have full sequence coverage, generally contain these dinoflagellate presequence features.

### *Symbiodinium* sp. C3 Membrane-Spanning Helix Organization

It has been hypothesized the Chl *a/b* and Chl *a/c* binding proteins evolved from a gene encoding four membrane-spanning helices [15]. It is proposed that the gene for the four membrane-spanning helices arose from gene duplication of membrane-spanning helices I and III with helix IV subsequently undergoing degeneration [15]. The conservation of helices I and III in a diverse range of organisms, including *Symbiodinium*, supports this hypothesis and that helices I and III and the associated Chl-binding residues represent the core of the light-harvesting complex [5], although alternative evolutionary scenarios have been suggested [4].

This report also demonstrates that *Symbiodinium* encodes at least two proteins with LHC membrane-spanning helix duplication, deletion, and/or degeneration; a feature more common in the Chl *a/b* binding proteins of green plants, chlorophytes and euglenophytes rather than Chl *a/c* and Fcp proteins. Comparison of the *Symbiodinium* acpPC sequences with LHCII genes from green plants [5] and LHCI genes in *Porphyridium cruentum* [9] demonstrates the similarity between them, particularly with respect to the identified Chl-binding residues. *Symbiodinium* has eight amino acids separating the two Chl-binding residues in helix II as documented in rhodophytes and chromophytes [9], rather than seven as in green plants [5]. While related to Chl *a/c* binding proteins, acpPCSym_17 and acpPCSym_KB8 A1.1_155 are particularly interesting and appear to have evolved independently from the polyproteins and Fcp binding proteins. Exhibiting 80% identity at the protein level, these two proteins are from different genetic strains of *Symbiodinium* [39], [74] and contain all the structural features and Chl-binding residues identified in the original green plant LHCII model [5]. Both encode three transmembrane helix and an amphiphilic α-helix protein, although helix II has eight amino acids between *Q* and *E* (fig. 3c).

The green plant LHCII sequence and structure [5], [6], [7] shares significant homology with LHC sequences from rhodophytes, crypotophytes, chromophytes and dinophytes. Superimposing rhodophyte and chromoalveolate sequences over the green plant LHCII model enables initial structural and Chl-binding site comparisons between and within different Chl-carotenoid binding protein groups, although definitive conclusions from such comparisons require caution. Superimposing the putative family of acpPC sequences onto the green plant LHCII model highlights the extent of sequence identity and suggests *Symbiodinium* may have a similar LHC structure to LHCII in green plants. The presence of *H. triquetra* and *K. micrum* sequences in the LI818 and LI818-like group and within the Chl *a/c* clades (Clade 1 and Clade 3a respectively) indicates the diversity shown within *Symbiodinium* is unlikely to have resulted from sample contamination during collection and extraction processes. The diversity of *Symbiodinium* LHC proteins poses the question of how the structure of the PSI and PSII supercomplexes is formed given such a range of LHCs in the *Symbiodinium* genome. In green plants, the PSI supercomplex is composed of two heterodimers consisting of *Lhca1-4*, with lower concentrations of *Lhca5-6*; in *Arabidopsis* these are each encoded by a single gene copy [75]. PSI in *Chlamydomonas* is more complex with up to 18 LHC isoforms being present, although all of these are homologous to the six LHC proteins found in green plants with the diversity arising from multiple genes for some of the LHC classes being present in the genome. The green plant PSII also contains six LHC proteins that form either trimers or monomers. Given the small number of genes encoding LHC in the green lineage the question arises as to how the dinoflagellate photosystem supercomplexes are formed and regulated with such a diversity of LHC isoforms being expressed? Does PSI contain only acpPC proteins from Clade 1, as is the case with red algae Clade 1, while acpPCs in Clade 3 associate with PSII? What role do acpPC isoforms with non-canonical Chl-binding residues play - are they still capable of chlorophyll binding or do they play an adapted role in photoprotection? These questions require further research. However, whatever their function, the range and complexity of *Symbiodinium* integral LHC proteins presented in this work, provides further evidence that the organization of dinoflagellate light-harvesting systems is unique [10].

## Supporting Information

Figure S1 **Northern blot analysis of freshly isolated** ***Symbiodinium*** **C3 and cultured** ***Symbiodinium*** **C1 RNA.** The blot was probed with sequence specific PCR product for acpPCSym_8 (lane 2 and 3), acpPCSym_13 (lane 4 and 5), acpPCSym_1 (lane 6 and 7), acpPCSym_4 (lane 8 and 9) and acpPCSym_15 (lane 10 and 11) labelled using dATP 5′ – [α-^32^P]. Equal quantities (2.5 µg) of RNA from *Symbiodinium* C3 and C1 were applied. Lanes 1 and 12 contain RNA standard probed with lambda DNA.
(DOCX)

Table S1 **Light-harvesting complex sequences from GenBank, NCBI, Swiss-Prot, PIR and EST databases.**
(DOCX)
