# Supplementary material for: Hyperdiversity of Genes Encoding Integral Light-Harvesting Proteins in the Dinoflagellate Symbiodinium sp
Source: PLoS One. 2012 Oct 24;7(10):e47456. doi: 10.1371/journal.pone.0047456 (PMC3480386; doi:10.1371/journal.pone.0047456)
Supplement: Figure S1 — Northern blot analysis of freshly isolated Symbiodinium C3 and cultured Symbiodinium C1 RNA. The blot was probed with sequence specific PCR product for acpPCSym_8 (lane 2 and 3), acpPCSym_13 (lane 4 and 5), acpPCSym_1 (lane 6 and 7), acpPCSym_4 (lane 8 and 9) and acpPCSym_15 (lane 10 and 11) labelled using dATP 5′ – [α-32P]. Equal quantities (2.5 µg) of RNA from Symbiodinium C3 and C1 were applied. Lanes 1 and 12 contain RNA standard probed with lambda DNA. (DOCX) [file pone.0047456.s001.docx]

**
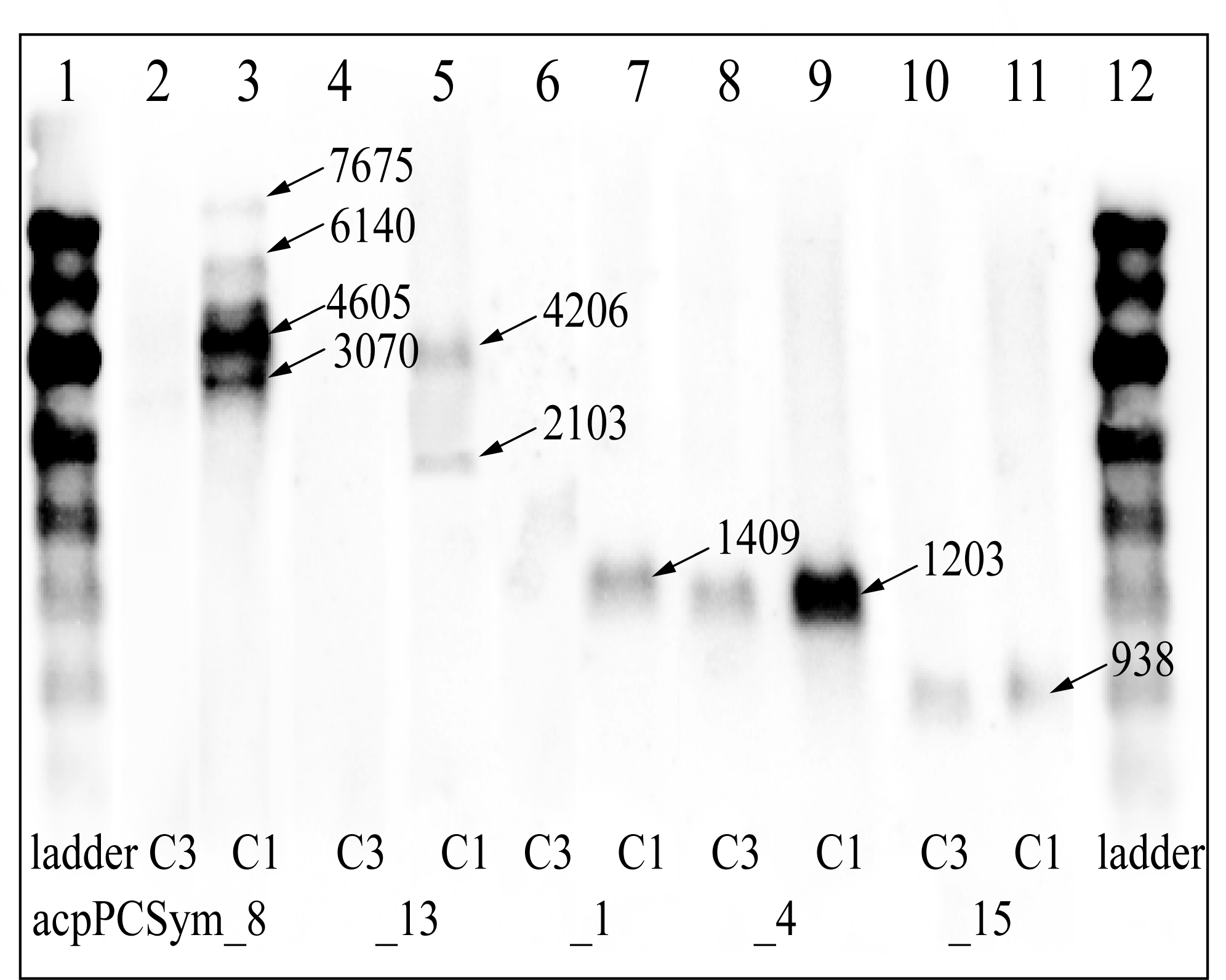
**

**Figure S1.** Northern blot analysis of freshly isolated *Symbiodinium* C3 and cultured *Symbiodinium* C1 RNA. The blot was probed with sequence specific PCR product for acpPCSym_8 (lane 2 and 3), acpPCSym_13 (lane 4 and 5), acpPCSym_1 (lane 6 and 7), acpPCSym_4 (lane 8 and 9) and acpPCSym_15 (lane 10 and 11) labelled using dATP 5’ – [α-^32^P]. Equal quantities (2.5 µg) of RNA from *Symbiodinium* C3 and C1 were applied. Lanes 1 and 12 contain RNA standard probed with lambda DNA.
