# Supplementary material for: Hyperdiversity of Genes Encoding Integral Light-Harvesting Proteins in the Dinoflagellate Symbiodinium sp
Source: PLoS One. 2012 Oct 24;7(10):e47456. doi: 10.1371/journal.pone.0047456 (PMC3480386; doi:10.1371/journal.pone.0047456)
Supplement: Table S1 — Light-harvesting complex sequences from GenBank, NCBI, Swiss-Prot, PIR and EST databases. (DOCX) [file pone.0047456.s002.docx]

Table S1 Light-harvesting complex sequences from GenBank, NCBI, Swiss-Prot, PIR and EST databases

| Molecular Database | Organism and Sequence | Accession Number |
| --- | --- | --- |
| GenBank | *Solanum lycopersicum* Lhca1, Lhca2, Lhca3 | AAA34140, CAA32197, CAA33330 |
|  | *Solanum lyocpersicum* Lhcb1*1, Lhcb5, Lhcb6a | AAA34147, CAA43590, AAA34143 |
|  | *Polystichum munitum* Lhcb | AAA68425 |
|  | *Chlamydomonas moewusii* Lhcb | CAA38635 |
|  | *Zea mays* Lhcb4 | CAA90681 |
|  | *Pinus sylvestris* Lhcb1*2 | CAA32658 |
|  | *Pisum sativum* Lhcb1 | CAA40365 |
|  | *Lemna gibba* Lhcb2 | AAA33396 |
|  | *Chlamydomonas reinhardtii* Lhcb | AAO16493 |
|  | *Dunaliella salina* Lhcb | AAA33278 |
|  | *Arabidopsis thaliana* Lhcb4 | CAA50712 |
|  | *Volvox carteri* Lhca | AAB40979 |
|  | *Chlamydobotrys stellata* Lhca | CAA50763 |
|  | *Chlamydomonas reinhardtii* Lhca I20 | CAA46235 |
|  | *Mantoniella squamata* Lhc | CAA49271 |
|  | *Euglena gracilis* Lhca1, Lhca2, Lhca5 | DAA05888, DAA05887, ABW06947 |
|  | *Guillardia theta* Lhc10, Lhc13 | CAM33413, AAF81522 |
|  | *Phaedactylum tricornutum* FcpB | CAA80897 |
|  | *Porphyridium cruentum* Lhca1, Lhca2 | AAB39488, AAB39489 |
|  | *Laminaria saccharina* Fcp | AAG13008 |
|  | *Saccharina japonica* Fcp | ACE80197 |
|  | *Heterosigma carterae* Fcp1 | CAA68028 |
|  | *Macrocystis pyrifera* Fcp3 | AAC49017 |
|  | *Pyrocystis lunula* Cac | AF508261 |
|  | *Karlodinium micrum* Cac1, Cac2 | ABI14390, ABA55565 |
|  | *Heterocapsa triquetra* Cac1, Cac2, Cac3, Cac5:2 | AAW79361, AAW79364, AAW79365, AAW79366 |
|  | *Amphidinium carterae* Cac | CAA08771 |
|  | *Bigelowiella natans* LI818, LI818*1 | DAA05890, AAP79202 |
|  | *Mesostigma viride* LI818 | DAA05932 |
|  | *Micromonas pusilla* LI818 | EEH60415 |
|  | *Gymnochlora stellate* LI818 | ACF24539 |
| NCBI | *Thalassiosira pseudonana* LI818, LI818*1 | XP_002295258, XP_002287075 |
|  | *Zea mays* Lhcb6, Lhcb5 | NP_001105375, NP_001105698 |
| Swiss-Prot | *Odontella sinensis* Fcp | Q42395 |
|  | *Isochrysis galbana* Fcp | Q39709 |
|  | *Dunaliella tertiolecta* Lhcb1 | P27517 |
| PIR | *Giraudyopsis stellifer* Cac | S60048 |
| EST | *Symbiodinium* sp. KB8 A1.1_155, _467 | FE539302, FE539303, FE538572 |
